# Intestinal Microbiota and Host Cooperate for Adaptation as a Hologenome

**DOI:** 10.1128/msystems.01261-21

**Published:** 2022-01-11

**Authors:** Hao Zhou, Lingyu Yang, Jinmei Ding, Ronghua Dai, Chuan He, Ke Xu, Lingxiao Luo, Lu Xiao, Yuming Zheng, Chengxiao Han, Fisayo T. Akinyemi, Christa F. Honaker, Yan Zhang, Paul B. Siegel, He Meng

**Affiliations:** a Department of Animal Science, School of Agriculture and Biology, Shanghai Jiao Tong University, Shanghai, People’s Republic of China; b Department of Animal and Poultry Sciences, Virginia Tech, Blacksburg, Virginia, USA; c Carilion Clinic, Roanoke, Virginia, USA; University of Connecticut

**Keywords:** Artificial selection, body weight, ceca, coevolution, DNA methylation, gut microbiota, holobiont, hologenome, microRNA

## Abstract

Multiomic analyses reported here involved two lines of chickens, from a common founder population, that had undergone long-term selection for high (HWS) or low (LWS) 56-day body weight. In these lines that differ by around 15-fold in body weight, we observed different compositions of intestinal microbiota in the holobionts and variation in DNA methylation, mRNA expression, and microRNA profiles in the ceca. Insulin-like growth factor 2 mRNA-binding protein 1 (*IGF2BP1*) was the most upregulated gene in HWS ceca with its expression likely affected by the upregulation of expression of gga-miR-2128 and a methylated region near its transcription start site (388 bp). Correlation analysis showed that *IGF2BP1* expression was associated with an abundance of microbes, such as *Lactobacillus* and *Methanocorpusculum.* These findings suggest that *IGF2BP1* was regulated in the hologenome in adapting to long-term artificial selection for body weight. Our study provides evidence that adaptation of the holobiont can occur in the microbiome as well as in the epigenetic profile of the host.

**IMPORTANCE** The hologenome concept has broadened our perspectives for studying host-microbe coevolution. The multiomic analyses reported here involved two lines of chickens, from a common founder population, that had undergone long-term selection for high (HWS) or low (LWS) 56-day body weight. In these lines that differ by around 15-fold in body weight, we observed different compositions of intestinal microbiota in the holobionts, and variation in DNA methylation, mRNA expression, and microRNA profiles in ceca. The insulin-like growth factor 2 mRNA-binding protein 1 (*IGF2BP1*) was the most upregulated gene in HWS ceca with its expression likely affected by a methylated region near its transcription start site and the upregulation of expression of gga-miR-2128. Correlation analysis also showed that *IGF2BP1* expression was associated with the abundance of microbes, such as Lactobacillus and *Methanocorpusculum*. These findings suggest that *IGF2BP1* was regulated in the hologenome in response to long-term artificial selection for body weight. Our study shows that the holobiont may adapt in both the microbiome and the host's epigenetic profile.

## INTRODUCTION

Although long-term artificial selection can alter the frequency of host genes and variation in its genome, the host genome is highly conserved, and genetic alterations within it are slow and rare. The response to selection may also depend on interactions between the host and microbiome. Phenotypic changes in the host may be the result of alterations of the hologenome, which is the sum of the genetic information of the host and its symbiotic microbes (1, 2). In this theory, the genomes of the holobiont act in a consortium rather than in isolation and function as a distinct evolutionary entity. Specifically, when subjected to selection, the evolution of a holobiont involves not only changes in the genome of the host but also a possible shift in microbiota. The microbes may allow the host to quickly adapt to the artificial selection (3, 4).

The synergistic phenotypes shaped for the holobiont under evolutionary forces involve host-microbe and intermicrobial collaborations (1). Examples include symbiotic microbiota that interact with their host and, thus, contribute to traits of the holobiont, such as obesity (5, 6), development (7), temperature adaptation (8), and immunity (9). Recently, researchers observed, in a long-term evolutionary study of Nasonia vitripennis, that atrazine exposure over 85 generations mediated adaptive changes within the microbiome and host genome, and the evolved microbiome metabolized atrazine more efficiently, which conferred host resistance to atrazine toxicity (10). In addition, the genetic selection of tropical tilapias for cold tolerance shaped the microbiome composition and modulated the hosts’ response to temperature (11). These findings imply that the host and its microbiota are not isolated from their counterpart in evolution but are interconnected and coregulated systems.

**FIG 1:**
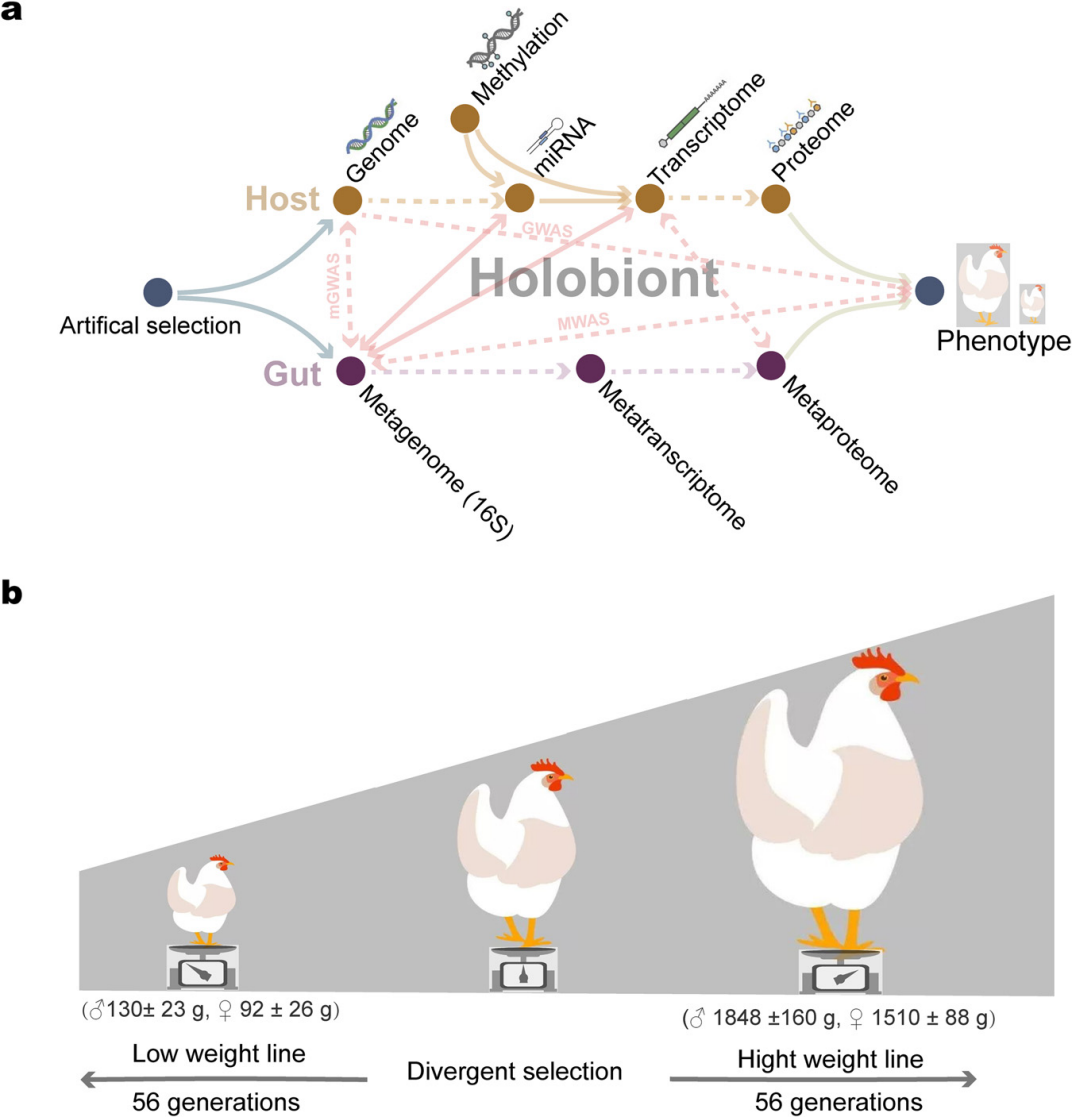
The holo-omic interactions in the holobiont and chicken model. (A) The holo-omic interactions between the host and its intestinal microbiota. Biomolecular interactions between hosts and symbiotic microorganisms triggered by artificial selection yield different holobiont phenotypes. Arrows indicate the directionality of the effect. Analyses performed in our study are shown by solid arrows, while dotted arrows represent associated relationships from other studies. Genome-wide association studies (GWAS), metagenome-wide association studies (MWAS), and metagenome genome-wide association studies (mGWAS). (B) Illustrations of chickens used in our study. The 56-day body weight of HWS and LWS males (♂) and females (♀) are presented in parentheses.

To date, coevolutionary mechanisms of the intestinal microbiota involved in responses of the host to selection have not been fully explored on hologenome epigenetic profiles in higher organisms. To study intimate biological cooperation between animals and their associated intestinal microbiota during long-term selection, it is necessary to apply a hologenome framework that integrates molecular data (Fig. 1A). Here, we report an experiment with the chicken as the model organism. The chickens were from lines that originated from a common founder population (12, 13) and were divergently selected for a single trait (56-day body weight) for 56 generations (Fig. 1B). Divergent selection resulted in a greater than 15-fold difference in body weight between the high (HWS) and low (LWS) weight lines at selection age (Fig. 1B). Using these chickens, we integrated molecular data of transcriptome, epigenome, and metagenome derived from either the host or symbiotic microorganisms into an integrative multiomic framework (Fig. 1A). The results suggest that the hologenome, which includes the microbiome composition and the epigenetic profile of the host, can be dynamic and change in response to artificial selection, thus leading to the evolution of the holobionts.

## RESULTS

### Characteristics of microbiota in the gastrointestinal tract.

Intestinal contents were collected from the duodenum, jejunum, ileum, ceca, and colon, respectively. A total of 85 high-quality samples were obtained from 10 HWS and 10 LWS chickens (Table S1). The microbiome of each segment from all 20 chickens was analyzed with 16S rRNA sequencing. A total of 8,918,125 reads with an average of 104,919 filtered reads per sample were yielded from high-throughput sequencing. The length distribution of the sample sequences was 226 (Fig. S1A). The rarefaction curve for each segment was made based on the obtained OTU data and showed that the sequencing depth was satisfied for the demand of analysis (Fig. S1B to F). These operational taxonomic units (OTUs) were generated and characterized for different taxonomic levels, including domain, phylum, class, order, family, and genus based on the Greengene database using QIIME. Taxonomies present in at least ¼ of the total samples were considered common, and their relative abundance counts were used for further analysis. A total of 21 phyla, 30 classes, 54 orders, 98 families, and 171 genera were characterized in these samples.

10.1128/mSystems.01261-21.2 FIG S1 (A) The length distribution of reads. (B to F) Rarefaction curves for OTUs of the duodenum, jejunum, ileum, ceca, and colon samples, respectively. Download FIG S1, TIF file, 1.8 MB. Copyright © 2022 Zhou et al. 2022 Zhou et al. https://creativecommons.org/licenses/by/4.0/ This content is distributed under the terms of the Creative Commons Attribution 4.0 International license.

10.1128/mSystems.01261-21.6 TABLE S1 Information of HWS and LWS lines and samples of HWS and LWS lines by trait, intestinal segment, and sex. Download Table S1, XLSX file, 0.02 MB. Copyright © 2022 Zhou et al. 2022 Zhou et al. https://creativecommons.org/licenses/by/4.0/ This content is distributed under the terms of the Creative Commons Attribution 4.0 International license.

**FIG 2:**
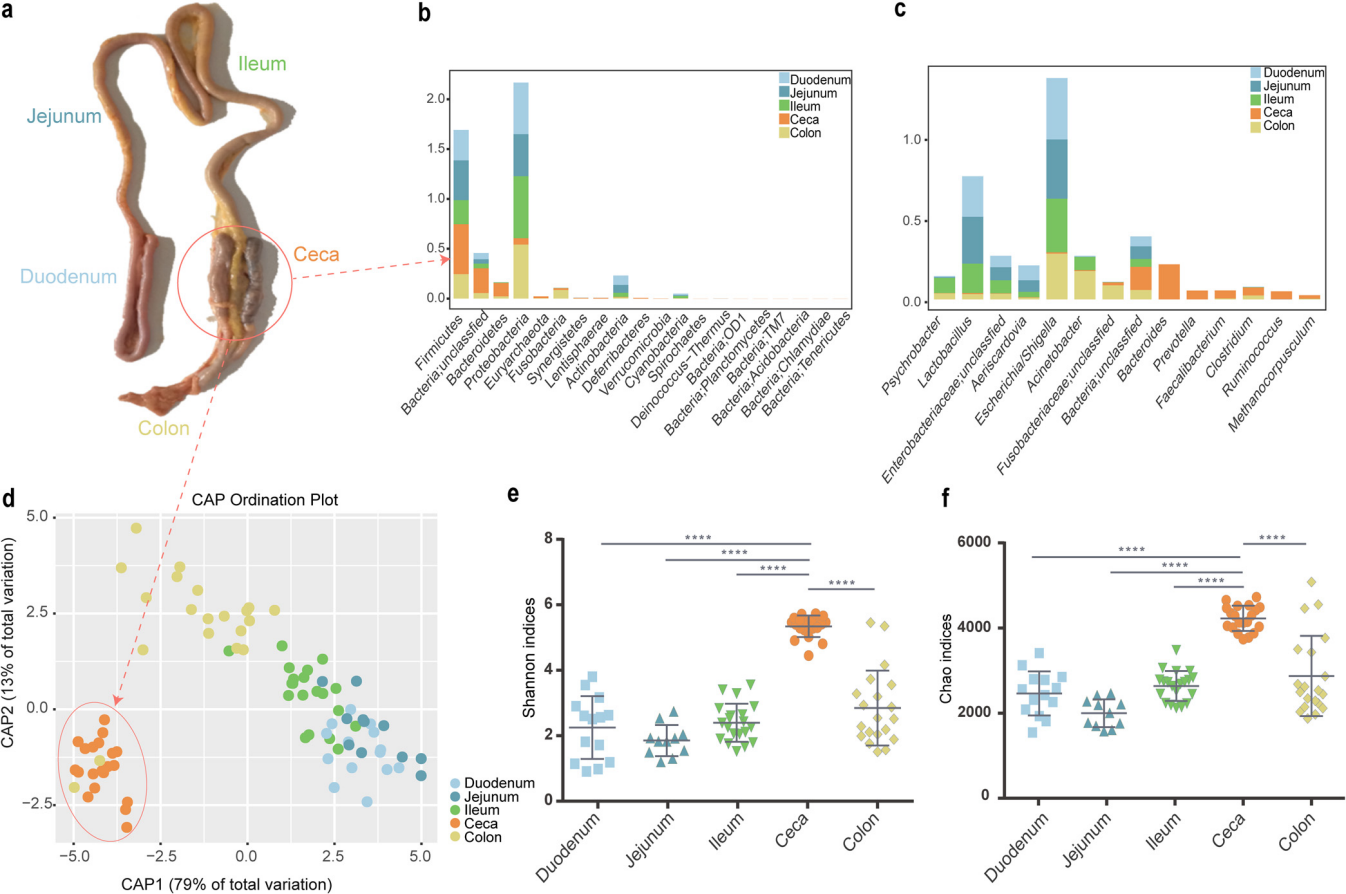
Composition and abundance of microbiota in the gastrointestinal tract. (A) Anatomy of the chicken intestinal tract. (B) Relative abundance of microbes at the phylum level among intestinal segments. (C) Relative abundance of microbes at the genus level among intestinal segments. (D) Canonical analysis of principal coordinates based on unweighted UniFrac metrics for the intestinal segments. (E) Shannon and (F) Chao1 methods were used for alpha diversity analysis of the microbiota of the intestinal segments. Quadruple asterisk (****) denotes *P* < 0.0001.

We then compared the distribution of intestinal microbiota in five different gastrointestinal (GI) segments (duodenum, jejunum, ileum, ceca, and colon) and found that Proteobacteria, Firmicutes, and Bacteroidetes were the 3 most dominant phyla, accounting for 80% of the microbiota (Fig. 2B and Table S2). However, these phyla had a dynamic pattern that differed among gastrointestinal segments. In intestinal segments (duodenum, jejunum, ileum, and colon), Proteobacteria was the most dominant phylum (>42%), followed by Firmicutes (>24%) and Bacteroidetes (<2%) (Fig. 2B and Table S2). Conversely, in the ceca, Proteobacteria accounted for only 6%, Bacteroidetes replaced Proteobacteria to become the most dominant phylum (40%), and the proportion of Firmicutes increased to 13% (Fig. 2B and Table S2). There was a high proportion of Euryarchaeota in the ceca (2.4%). In addition, 14% of the OTUs belonging to the Bacteria domain were annotated as unclassified (Fig. 2B and Table S2).

10.1128/mSystems.01261-21.7 TABLE S2 The abundance of phyla and genera in intestinal segments. Download Table S2, XLSX file, 0.01 MB. Copyright © 2022 Zhou et al. 2022 Zhou et al. https://creativecommons.org/licenses/by/4.0/ This content is distributed under the terms of the Creative Commons Attribution 4.0 International license.

We also measured the composition of intestinal microbiota at the genus level and identified the 5 main genera of Bacteria and the main genus of Archaea. A total of 14 common genera were identified (Fig. 2C and Table S2). In the ceca, *Bacteroides* (21%), *Prevotella* (5%), *Faecalibacterium* (4.9%), *Clostridium* (4.9%), and *Ruminococcus* (4.8%) were the most abundant genera. *Methanocorpusculum* accounted for 2.1%. For intestinal segments, *Escherichia/Shigella* was the most enriched genus (>28%), followed by *Lactobacillus* (>18% for small intestinal segments) and *Acinetobacter* (17% for colon). Taken together, these data demonstrated that the preponderant microbes differed among intestinal segments and reflect diverse functions among gastrointestinal segments.

The canonical analysis of principal coordinates (CAP), a method that measures phylogenetic dissimilarities between microbial communities based on Unifrac metrics, showed a separation between samples from the ceca and other sections of the GI tract (Fig. 2D). Samples from the duodenum, jejunum, ileum, and colon tended to cluster with each other. Furthermore, Shannon, ACE, Simpson, and Chao1 analyses for alpha diversity showed greater diversity and abundance of cecal microbiota than other intestinal segments (*P* < 0.0001, Tukey's multiple-comparison test; Fig. 2E and F and Table S3). Correlation coefficients between the duodenum, jejunum, and ileum were high, ranging from 0.56 to 0.95 (*P* < 0.05) and less than the 0.14 (*P* < 0.05) for those between the ceca and these intestinal segments (Table S4). These results, when taken together, demonstrate that the microbiota of the ceca is considerably more diverse than those of the small intestinal segments.

10.1128/mSystems.01261-21.8 TABLE S3 Comparisons of alpha diversity indices of intestinal microbiota among intestinal segments. Download Table S3, XLSX file, 0.01 MB. Copyright © 2022 Zhou et al. 2022 Zhou et al. https://creativecommons.org/licenses/by/4.0/ This content is distributed under the terms of the Creative Commons Attribution 4.0 International license.

10.1128/mSystems.01261-21.9 TABLE S4 Correlations among intestinal segments for HWS and LWS. Download Table S4, XLSX file, 0.1 MB. Copyright © 2022 Zhou et al. 2022 Zhou et al. https://creativecommons.org/licenses/by/4.0/ This content is distributed under the terms of the Creative Commons Attribution 4.0 International license.

### The alteration of intestinal microbiota under artificial selection.

**FIG 3:**
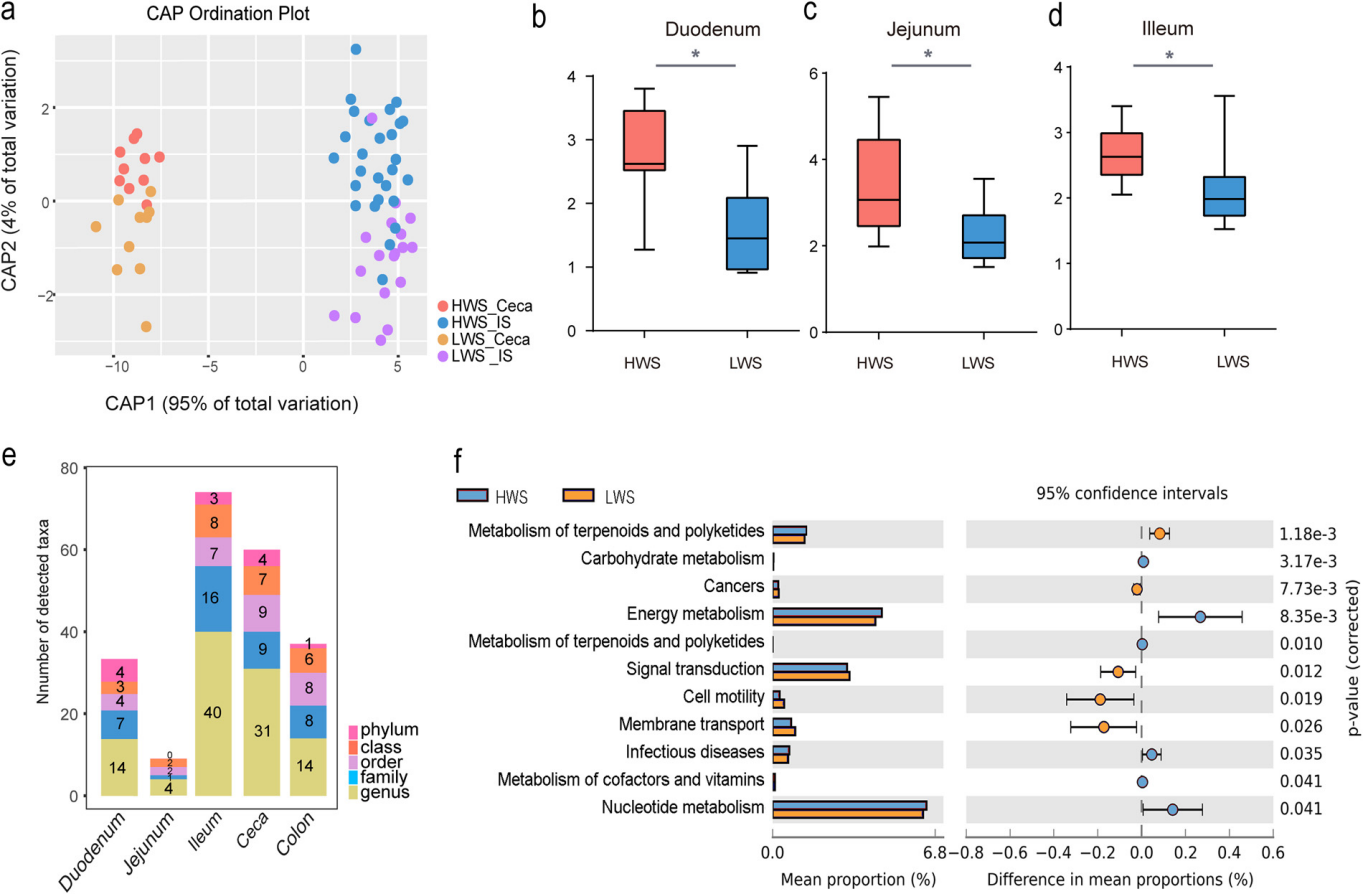
Altered microbiome in gastrointestinal tract under artificial selection. (A) Canonical analysis of principal coordinates based on unweighted UniFrac metrics between ceca and other intestinal segments (duodenum, jejunum, ileum, and colon) of HWS and LWS. (B to D) Comparisons of alpha diversity between HWS and LWS with the Shannon method. Single asterisk (*) denotes *P* < 0.05. (E) Significantly different abundances of microbes observed at each classification level for each intestinal segment (LDA > 2, *P* < 0.05). (F) Comparisons of functional microbial pathways in HWS and LWS.

Canonical analysis of principal coordinates based on UniFrac metrics showed that samples from HWS and LWS ceca and other intestinal segments (duodenum, jejunum, ileum, and colon) formed distinct groups (Fig. 3A). Adonis, a nonparametric permutational multivariate analysis (*P* = 0.001, HWS versus LWS), and ANOSIM (analysis of similarities) test (*P* = 0.001) further confirmed significant differences between the HWS and LWS lines. In addition, according to the analysis of alpha diversity for Shannon indices, small intestinal segments (duodenum, jejunum, and ileum) were significantly different (*P* < 0.05, Student's *t* test) between the two lines with alpha diversity substantially less in LWS than HWS (Fig. 3B to D). The alpha diversity of ceca (mean 4.82 versus 5.25, *P* = 0.13) and colon (mean 1.79 versus 1.86, *P* = 0.85) were also lower in LWS than HWS, but these differences were not significant. Overall, these results indicate that the composition of the intestinal microbiota of LWS and HWS chickens are different.

To further measure the variation of intestinal microbiota in the intestinal segments between these lines, comparisons were made at the phylum, class, order, family, and genus levels. The difference in abundance of microbes for HWS and LWS at each classification level is shown in Fig. 3E. The ceca and ileum had the greatest variation in the number of taxa, suggesting that the species of microbes that lived in these segments were more affected by selection for high or low body weight than those in the other intestinal segments. The difference between the microbiota of HWS and LWS jejuna was the least. At the phylum level, only Proteobacteria and Firmicutes abundance were significantly increased in the duodenum and ceca in LWS. In HWS, Euryarchaeota was significantly enriched in the ileum and ceca (Fig. S3).

10.1128/mSystems.01261-21.4 FIG S3 (A) Differentially abundant microbes identified by Lefse at the phylum level. Download FIG S3, TIF file, 2.6 MB. Copyright © 2022 Zhou et al. 2022 Zhou et al. https://creativecommons.org/licenses/by/4.0/ This content is distributed under the terms of the Creative Commons Attribution 4.0 International license.

The greatest number of microbes changed at the genus level, which has a relatively precise taxonomy. When we compared the microbes in each segment of HWS and LWS, of 199 genera, 31 were significantly different in the ceca (*P* < 0.05, Table 1). Among these, only the abundance of *Collinsella* was greater in LWS than HWS, while the other 30 genera were greater in HWS than LWS. Notably, included in these was the abundant genus in the ceca, *Bacteroides*. In addition, 6 genera, *Faecalibacterium* (LDA = 3.56, *P* = 0.0002), *Sporobacter* (LDA = 3.07, *P* = 0.0003), *Oscillibacter* (LDA = 3.04, *P* = 0.0019), *Subdoligranulum* (LDA = 2.86, *P* = 0.0025), *Hydrogenoanaerobacterium* (LDA = 2.21, *P* = 0.0156), and *Butyricicoccus* (LDA = 2.11, *P* = 0.0102) belonging to the *Ruminococcaceae* family, and 2 genera, *Dorea* (LDA = 3.2, *P* = 0.0004) and *Anaerosporobacter* (LDA = 2.04, *P* = 0.0012), in *Lachnospiraceae* were significantly greater in HWS than LWS. The most abundant genus of Archaea, *Methanocorpusculum*, was also greater in HWS than LWS. Overall, the microbiota of the ceca in HWS were most dramatically different during selection for high or low body weight than the other intestinal segments.

**TABLE 1:** Genera that differed significantly in the ceca of high (HWS) and low (LWS) weight chicken lines

Phylum	Family	Genus	Group^*a*^	LDA^*b*^	*P* value
Actinobacteria	Actinomycetaceae	*Actinomyces*	HWS	2.79	0.0223
	Bifidobacteriaceae	*Aeriscardovia*	HWS	2.25	0.0002
	Propionibacteriaceae	*Propionibacterium*	HWS	2.59	0.0306
	Coriobacteriaceae	*Collinsella*	LWS	3.29	0.0306
		*Olsenella*	HWS	2.47	0.0002
Bacteroidetes	Bacteroidaceae	*Bacteroides*	HWS	2.10	0.0126
	Rikenellaceae	*Alistipes*	HWS	3.26	0.0012
	Porphyromonadaceae	*Parabacteroides*	HWS	2.86	0.0306
		*Butyricimonas*	HWS	2.07	0.0032
Firmicutes	Clostridiaceae	*Clostridium*	HWS	3.45	0.0007
	Eubacteriaceae	*Alkalibacter*	HWS	2.82	0.0047
	IncertaeSedisXIV	*Blautia*	HWS	2.17	0.0015
	Veillonellaceae	*Veillonella*	HWS	2.39	0.0002
	Lachnospiraceae	*Dorea*	HWS	3.20	0.0004
		*Anaerosporobacter*	HWS	2.04	0.0012
	Lactobacillaceae	*Lactobacillus*	HWS	2.91	0.0002
	Ruminococcaceae	*Faecalibacterium*	HWS	3.56	0.0002
		*Sporobacter*	HWS	3.07	0.0003
		*Oscillibacter*	HWS	3.04	0.0019
		*Subdoligranulum*	HWS	2.86	0.0025
		*Hydrogenoanaerobacterium*	HWS	2.21	0.0156
		*Butyricicoccus*	HWS	2.11	0.0102
Proteobacteria	Caulobacteraceae	*Brevundimonas*	HWS	2.33	0.0464
	Helicobacteraceae	*Helicobacter*	HWS	3.09	0.0284
	Methylobacteriaceae	*Methylobacterium*	HWS	2.64	0.0265
	Moraxellaceae	Acinetobacter	HWS	2.84	0.0002
	Enterobacteriaceae	Escherichia *_Shigella*	HWS	2.79	0.0032
		*Thorsellia*	HWS	2.05	0.0130
Lentisphaerae	Victivallaceae	*Victivallis*	HWS	2.49	0.0413
Euryarchaeota	Methanocorpusculaceae	*Methanocorpusculum*	HWS	3.42	0.0002
Synergistetes	Synergistaceae	*Cloacibacillus*	HWS	2.70	0.0009

a Group: The line with the significantly greater relative abundance.

b LDA: Linear discriminant analysis.

Similar to the ceca, the microbiota of the ileum differed between the lines with 40 genera being significantly different (*P* < 0.05, Table S5). Among them, the abundance of 14 genera was greater in HWS than LWS, while 26 genera were greater in LWS than HWS. *Psychrobacter*, the microbe with the highest LDA effect (LDA = 4.70), increased in the LWS ileum. However, in the duodenum, 14 genera differed between the lines with 12 significantly greater in HWS than LWS. Although *Lactobacillus* was the genus with the greatest abundance in HWS with an LDA score of 5.31, the abundance of *Escherichia/Shigella* and *Pseudobutyrivibrio* were significantly greater in LWS than HWS (Table S5). In contrast, there were only 4 microbes with significantly different abundance between the lines in the jejunum. These were *Ruminococcus* (LDA = 2.09, *P* = 0.03), *Victivallis* (LDA = 2.16, *P* = 0.03), *Alistipes* (LDA = 2.07, *P* = 0.03), and *Turicibacter* (LDA = 2.11, *P* = 0.03). Colon microbes differed between HWS and LWS for 14 genera (*P* < 0.05, Table S5). Among them, 6 had greater abundance in HWS than LWS with LDA scores for 3 genera greater than 4 (*Lactobacillus*, [LDA = 4.57, *P* = 0.001], *Faecalibacterium* [LDA = 4.38, *P* = 0.041], and *Aeriscardovia* [LDA = 4.23, *P* < 0.001]). In contrast, 9 genera had higher abundance in LWS than HWS with LDA scores for 3 genera being greater than 4 (*Escherichia/Shigella* [LDA = 4.13, *P* = 0.019], *Sporosarcina* [LDA = 4.76, *P* = 0.019], and *Paenibacillus* [LDA = 4.39, *P* = 0.04]).

10.1128/mSystems.01261-21.10 TABLE S5 Different microbes in duodenum, jejunum, ileum, ceca, and colon of HWS and LWS. Download Table S5, XLSX file, 0.02 MB. Copyright © 2022 Zhou et al. 2022 Zhou et al. https://creativecommons.org/licenses/by/4.0/ This content is distributed under the terms of the Creative Commons Attribution 4.0 International license.

### The association of cecal microbial function with body weight.

When we analyzed the whole intestinal microbial genome at the broadest level, 11 pathways differed significantly between the lines (Fig. 3F). Enriched markers were frequently associated with the functional pathways of metabolism of terpenoids and polyketides, carbohydrate metabolism, energy metabolism, infectious diseases, metabolism of cofactors and vitamins, and nucleotide metabolism in HWS. In contrast, the cecal microbiota of LWS exhibited enrichment in membrane transport, cancers, signal transduction, and cell motility. Moreover, the pathways associated with carbohydrate and energy metabolism were significantly enriched in HWS (Fig. 3F). When the more focused subsystem levels were analyzed, 61 pathways were significantly different between the lines (see Table S6 at https://doi.org/10.6084/m9.figshare.16830736.v7). Among them, 5 (glycolysis/gluconeogenesis, citric acid cycle [TCA cycle], butanoate metabolism, amino sugar, and nucleotide sugar metabolism, and pyruvate metabolism) are involved in carbohydrate metabolism. Three pathways linked to energy metabolism (oxidative phosphorylation, methane metabolism, and carbon fixation pathways in prokaryotes) were enriched in HWS. The PPAR signaling pathway was also enriched in HWS (see Table S6 at https://doi.org/10.6084/m9.figshare.16830736.v7). Taken together, microbial function analyses showed that HWS cecal microbiota presented higher activity associated with carbohydrate and energy metabolism than LWS. As such, they may have contributed, in part, to the large differences in body weight between these lines, which originated from a common founder population and were selected for the same trait, 56-day body weight.

### Variation in host gene expression under artificial selection.

**FIG 4:**
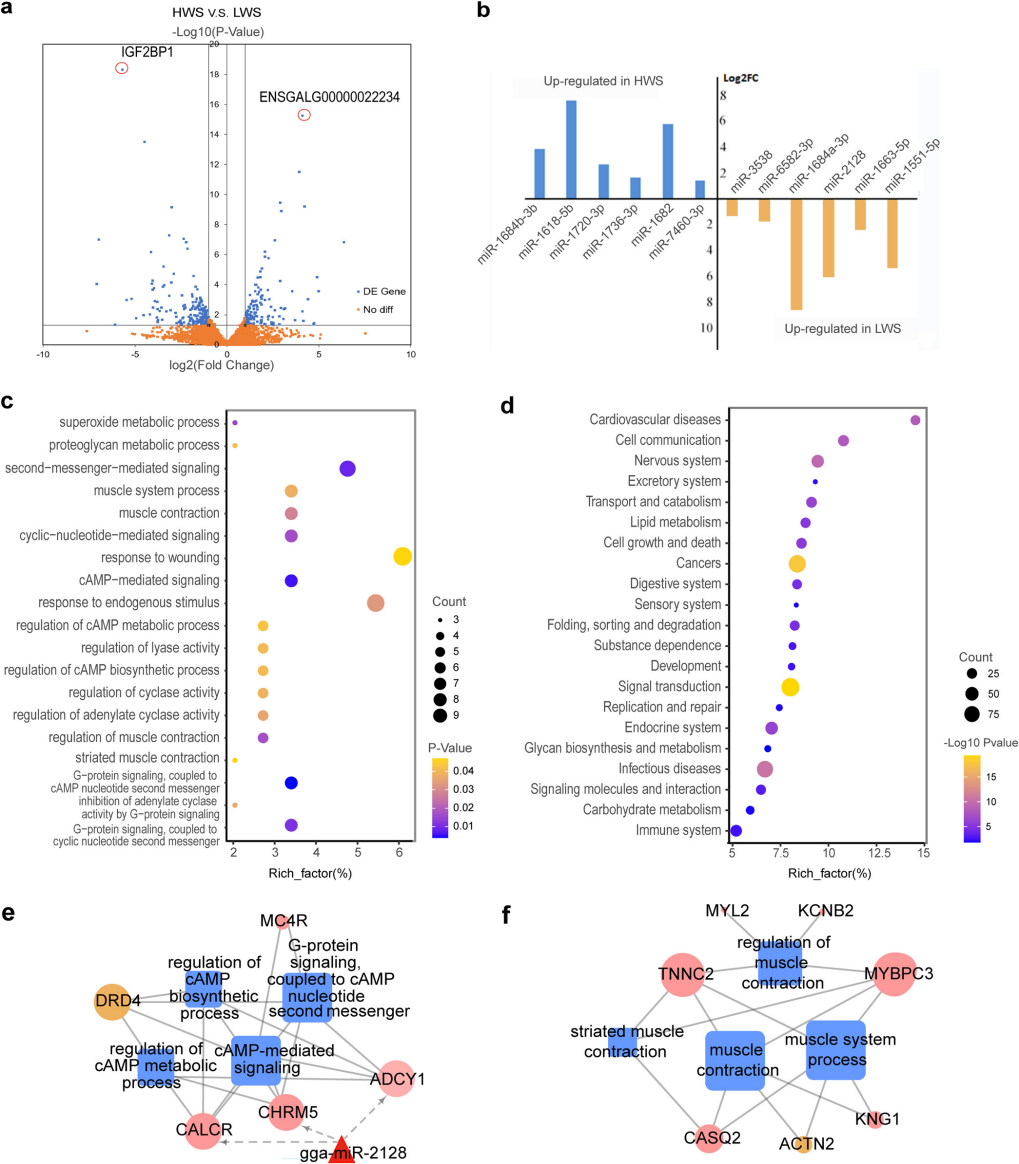
Variation in host cecal gene expression under artificial selection. (A) Volcano map of differentially expressed genes for HWS and LWS. (B) Bar plot shows the differentially expressed miRNAs for HWS and LWS. (C) Enrichment chart of GO terms enriched by significant differentially expressed genes between HWS and LWS. The rich factor was the ratio of the differential genes *versus* all genes involved in specific pathways. (D) Enrichment chart of pathway analysis of differentially methylated genes between HWS and LWS. (E and F) Network analysis of DEGs and their enriched functions, where triangles, squares, and circles represent miRNAs, go terms, and genes, respectively, pink and green nodes represent the upregulated and downregulated genes in HWS and LWS, respectively.

The results for mRNA-Seq are shown in Table S19 at https://doi.org/10.6084/m9.figshare.16830736.v7. There were 327 genes with >2-fold changes (*P* < 0.05), which we considered differentially expressed genes (DEGs). Of these, 198 were upregulated and 129 were downregulated in HWS compared with LWS (Fig. 4A, see Table S8 at https://doi.org/10.6084/m9.figshare.16830736.v7). The most significantly upregulated gene in HWS was *IGF2BP1* (fold change = 51.33, *P* = 5.02 × 10^−19^), and in LWS was *ENSGALG00000022234* (fold change = 17.47, *P* = 5.71 × 10^−16^) (Fig. 4A).

**FIG 5:**
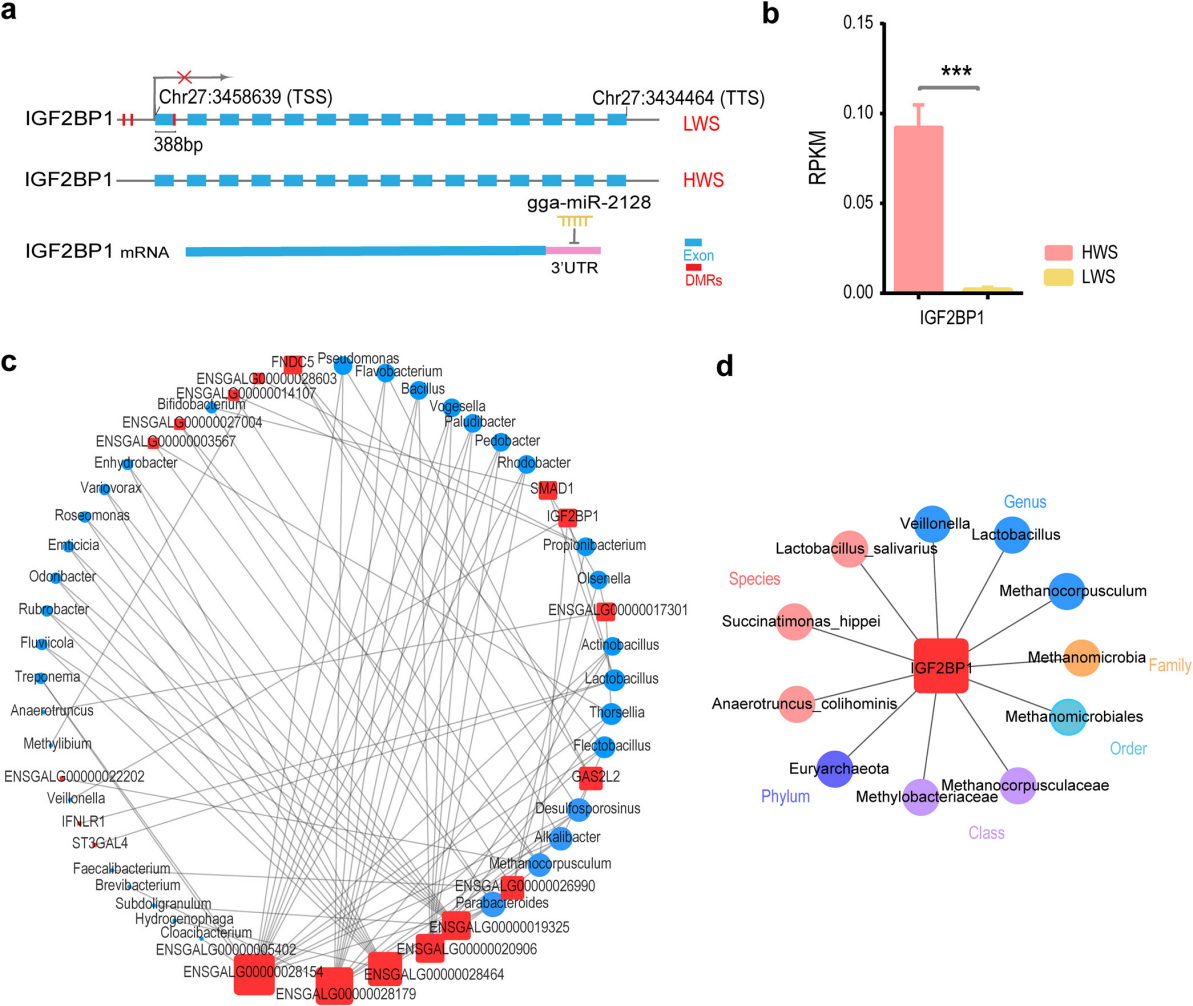
*IGF2BP1* interactions with intestinal microbes. (A) The regulation of *IGF2BP1* expression through methylation and miRNA. (B) Gene expression levels of *IGF2BP1* in HWS and LWS (*P* = 5e-19). The error bars show the SE of biological replicates (*n* = 10). (C) The interaction network between significantly upregulated genes and genera in the ceca. (D) The interaction network between *IGF2BP1* and intestinal microbes at each classification level.

To better understand the functions of DEGs, we analyzed the functional distribution of DEGs in the ceca of these chicken lines according to gene ontology (GO) enrichment and Kyoto Encyclopedia of Genes and Genomes (KEGG) pathways analyses. Of the 20 significantly enriched GO terms for biological processes, most were enriched in cyclic AMP (cAMP) and muscle-related functions (Fig. 4C, see Table S9 at https://doi.org/10.6084/m9.figshare.16830736.v7). The cAMP process included cAMP-mediated signaling and G-protein signaling coupled to a cAMP nucleotide second messenger, which regulates the cAMP biosynthetic and metabolic processes (Fig. 4C and E). In addition, *CALCR*, *ADCY1*, *CHRM5*, *MC4R*, which belong to cAMP-related functions, had greater expression in HWS than LWS (Fig. 4E). Moreover, muscle-related functions, which include regulation of muscle contraction, muscle system processes, and striated muscle contraction, were involved with 7 genes (Fig. 4C and F). Of them, 6 (*KNG1*, *TNNC2*, *MYBPC3*, *CASQ2*, *MYL2*, *KCNB2*) were greater in HWS (Fig. 4F). In addition, negative regulation of biosynthetic processes was significantly enriched, which included *IGF2BP1* that was upregulated in HWS (Fig. 5B). The KEGG pathways analysis showed that complement and coagulation cascades (*P* = 0.007) and neuroactive ligand-receptor interactions (*P* = 0.015) were the more enriched pathways (see Table S9 at https://doi.org/10.6084/m9.figshare.16830736.v7).

### Epigenetic changes under artificial selection.

The MEDIPS package was employed to quantify methylation levels, and edgeR was used to analyze the differentially methylated regions (DMR). Of the 4,779 DMRs throughout the whole genome, 3,430 and 1,349 were hypomethylated in HWS and LWS, respectively. The distribution of DMRs per chromosome (Fig. S2A) showed that chromosome 1, the largest chromosome in the chicken genome, accounted for 883 DMRs, and the sex chromosome W had the fewest DMRs with 3.

10.1128/mSystems.01261-21.3 FIG S2 (A) The distribution of DMRs on different chromosomes. (B) The length distribution of sequencing reads from small RNA-Seq. Download FIG S2, TIF file, 2.2 MB. Copyright © 2022 Zhou et al. 2022 Zhou et al. https://creativecommons.org/licenses/by/4.0/ This content is distributed under the terms of the Creative Commons Attribution 4.0 International license.

Of the 4,779 DMRs, 3,841 were located at intergenic regions and 938 were distributed in the promoter, exon, intron, 5′, 3′ untranslated region (UTR), and transcription termination sites (TTS), which were considered differentially methylated genes (DMGs) (see Table S11 at https://doi.org/10.6084/m9.figshare.16830736.v7). There were more DMRs in the exon and intron than in the promoter and other gene regions. More hypermethylated DMRs were distributed in an intron, exon, 3′ UTR, and TTS in HWS than LWS. In contrast, there were more hypermethylated DMRs in promoter and 5′ UTR in LWS than HWS. When we identified the genes that contained the DMRs, 437 and 204 hypermethylated genes were characterized in HWS and LWS, respectively (see Table S12 at https://doi.org/10.6084/m9.figshare.16830736.v7). The numbers of hypermethylated genes relating to promoter-TSS for HWS and LWS were 16 and 51, respectively (see Table S13 at https://doi.org/10.6084/m9.figshare.16830736.v7). Genes *SRP68*, *SOX9*, *PRKRIP1*, *PPP2R4*, and *PDCD2* were continuously hypermethylated for more than 2 DMRs in LWS, and the promoter-TSS location was included in hypermethylated regions. However, for HWS, only *RPL3L* was hypermethylated for more than 2 DMRs. In addition, the CpG island involved in DMRs was identified. Of 22 DMGs characterized (see Table S14 at https://doi.org/10.6084/m9.figshare.16830736.v7), all regions were hypomethylated in HWS. Functional analysis revealed that 641 DMGs were significantly enriched in 23 pathways (Fig. 4D). Involved were transport and catabolism, lipid metabolism, glycan biosynthesis and metabolism, and digestion.

We observed 345 mature miRNAs. Of them, 12 differentially expressed miRNAs (DEMs) were identified (Fig. 4B). Six (gga-miR-1684b-3p, gga-miR-1618-5p, gga-miR-1720-3p, gga-miR-1736-3p, gga-miR-1682, and gga-miR-7460-3p) were upregulated and 6 (gga-miR-1684a-3p, gga-miR-2128, gga-miR-1663-5p, gga-miR-1551-5p, gga-miR-3538, and gga-miR-6582-3p) were downregulated in HWS relative to their expression in LWS (Fig. 4B).

### Regulatory network analysis of DEMs and DEGs.

In HWS, the lower expressed DEMs consisted of gga-miR-2128 and gga-miR-1684a-3p and targeted 58 DEGs, which were upregulated (see Table S10 at https://doi.org/10.6084/m9.figshare.16830736.v7). Of the 58 DEGs, *CALCR*, *ADCY1*, and *CHRM5* were enriched in related cAMP functions (Fig. 4E). One gga-miR-2128 target was *IGF2BP1* (Fig. 5A). Biological process (BP) functions of O-glycan processing (*P* = 0.08) were associated with *MUC1* and *ST3GAL4* that were putatively regulated by gga-miR-2128. Moreover, the upregulated DEMs in HWS, including gga-miR-1682, gga-miR-1618-5p, and gga-miR-1684b-3p, targeted 44 DEGs whose expression was downregulated (see Table S10 at https://doi.org/10.6084/m9.figshare.16830736.v7). In addition, the BP functions of actin binding (*P* = 0.03) and motor activity (*P* = 0.06) were enriched by targeted DEGs, including *DNASE1*, *MYO3A*, *CSRP3*, and *KIF18B*.

### Integration analysis of DNA methylation data with DEGs.

Methylation of DNA in both promoter and gene coding regions is associated with gene expression (14). To identify potential functionally relevant DNA methylation changes, we integrated gene expression data and DNA methylation profiles in HWS and LWS. A total of 938 differentially methylated genes were selected to investigate concomitant changes in expression. Among the 327 DEGs, 16 were associated with changes in methylation (see Table S15 at https://doi.org/10.6084/m9.figshare.16830736.v7). Of these, *DRD4* expression was differentially downregulated in the HWS and hypermethylated in the region of TTS. The exon of *CSRP3* in HWS was hypermethylated and showed upregulated expression. The methylation of 13 genes (*FSTL4*, *SMAD1*, *EDNRB2*, LOC769357, *PRKAA2*, *HHATL*, *IGF2BP1*, *ST6GALNAC2*, *LRRTM3*, *ZPBP2*, *CD8B*, *F7*, and *KIFC1*) occurred in the intron region. The DMR location for *IGF2BP1* in LWS was involved in the CpG island, and its distance to TSS was only 388 bp (Fig. 5A) with the expression of *IGF2BP1* most differentially downregulated (Fig. 5B).

### The association between DEGs and intestinal microbiota in ceca.

To measure the associations between host and intestinal microbiota in the ceca, 19 upregulated and 19 downregulated DEGs in the HWS (FDR < 0.01, see Table S8 at https://doi.org/10.6084/m9.figshare.16830736.v7) and cecal microbes annotated at each level were analyzed together to form a correlation network. The focus on the genus level showed 90 and 37 edges between cecal microbes and upregulated genes in HWS and LWS, respectively (|r|>0.6, *P* < 0.01, Pearson method) (see Table S16 and S17 at https://doi.org/10.6084/m9.figshare.16830736.v7). In the network of upregulated DEGs in HWS, Growth Arrest Specific 2 Like 2 (*GAS2L2*) was connected to the *Alkalibacter* genus with the highest r being 0.8 (*P* = 0.00003) and the degree of *GAS2L2* and *Alkalibacter* in the network were the major 4 and 9, respectively (Fig. 5C). *Parabacteroides* and *Methanocorpusculum* were the nodes of genera with the top 1 and 2 degrees and were significantly more abundant in HWS (Fig. 5C and Table 1). *IGF2BP1* was connected to *Veillonella* (*r* = 0.65, *P* = 0.002), *Lactobacillus* (*r* = 0.62, *P* = 0.004), and *Methanocorpusculum* (*r* = 0.61, *P* = 0.004) (Fig. 5C and Table 1). In addition, the Lactobacillus salivarius species was positively correlated with *IGF2BP1* (*r* = 0.65, *P* = 0.002) (Fig. 5D). All these *IGF2BP1*-related microbes were highly enriched in HWS (Table S5).

For the correlation of upregulated DEGs in LWS and cecal microbes, *Collinsella* was significantly abundant and connected to upregulated *ENSGALG00000011906*. *Anaerotruncus* was the genus with the highest connected degree in all nodes, and the correlation of *Anaerotruncus* and *GFAP* was the highest in the 74 pairs of genera and upregulated genes (degree = 8, *r* = 0.73, *P* = 0.0003, see Table S17 at https://doi.org/10.6084/m9.figshare.16830736.v7). Overall, these findings demonstrated different patterns for the microbiota in HWS and LWS ceca. Moreover, several genes influenced the abundance of certain cecal microbes.

## DISCUSSION

The microbiota in the gastrointestinal tract have an important function in improving food absorption and boosting the immune system, thus influencing both host development and health (15, 16). Recent studies reported that the chicken microbiome varies in different intestinal segments (17–20). The most densely inhabited microbial community within the chicken intestine was observed in the ceca, a pair of blind-ended sacs that open at the junction between the small and large intestine (21). The ceca has an essential role in nutrition via the production of short-chain fatty acids, nitrogen recycling, and amino acid production, influencing both the health and development of chickens (20, 22). In the present study, the structures and composition of intestinal microbiota distributed in the duodenum, jejunum, ileum, ceca, and colon from HWS and LWS chickens were investigated. Observed were large differences in microbiota between samples obtained from the ceca and those from other intestinal segments. Alpha diversity analysis showed greater diversity and richness of cecal microbiota than in the duodenum, jejunum, ileum, ceca, and colon. In addition, we found that while *Bacteroides*, *Prevotella*, *Faecalibacterium*, and *Ruminococcus* were the predominant genera in ceca, their presence was rarer in other intestinal segments. Similar findings were reported in studies performed in commercial broilers (17) and layers (18, 20). *Bacteroides* spp. are recognized to degrade a wide range of ordinarily indigestible dietary plant polysaccharides, such as amylose, amylopectin, and pullulan (23, 24), and *Ruminococcus* is linked to polysaccharide degradation and utilization in chicken intestines (17). *Bacteroides* and *Faecalibacterium* have been suggested to influence the suppression of regulatory T cell expansion and the promotion of anti-inflammatory cytokine production (25–27). These findings reveal roles that *Bacteroides*, *Ruminococcus*, and *Faecalibacterium* have in the growth and health of chickens. These characteristics infer that the ceca are an integral area for host-microbe interactions and coadaptation research.

A diverse distribution of cecal microbiota was observed between lines of chickens that had undergone long-term divergent selection for high and low body weight at 56 days of age. These lines, HWS and LWS, originated from a common founder population and were fed common diets throughout. CAP analysis showed that the intestinal microbiota were significantly influenced by the selection for high or low juvenile body weight. Six genera (*Faecalibacterium*, *Sporobacter*, *Oscillibacter*, *Subdoligranulum*, *Hydrogenoanaerobacterium*, and *Butyricicoccus*) from the *Ruminococcaceae* family and 2 genera (*Dorea* and *Anaerosporobacter*) from the *Lachnospiraceae* family were greater in HWS than LWS. These bacteria possess abundant and diverse glycoside hydrolases (GH) and carbohydrate-binding modules (CBM), which can decompose cellulose and hemicellulose components of plant material that are indigestible by the host. These compounds are then fermented and converted into short-chain fatty acids (SCFAs) (mainly butyrate, acetate, and propionate) that can be absorbed and used for energy by the host (28). For animals who prefer fiber diets, SCFAs are the important energy substrate for metabolic energy production (29). High SCFA content produced by intestinal microbiota are thought to have a key role in increasing the capacity of the host to utilize excess energy from the diet, which can promote weight gain. This has been reported in the chickens, piglets, rabbits, and calves (30–33). In addition, humans with high *Prevotella*-to-*Bacteroides* ratios have lower body weight and less body fat than those with low *Prevotella*-to-*Bacteroides* ratios (34, 35), indicating that the proportion of *Bacteroides* may be associated with changes in body fat and muscle. In our study, *Bacteroides* were significantly more abundant in HWS than LWS, while the distribution of *Prevotella* was the same in both lines. Taken together, these results showed that the selection for high or low body weight dramatically altered the composition of microbiota in the intestine. In essence, these altered microbes, through variation of microbial amplification or reduction modifies the composition of the hologenome, which may contribute to the regulation of the host’s growth. The functional analysis of the intestinal microbiota of HWS and LWS provides additional support for this conclusion.

The TCA cycle is an important metabolic pathway that coordinates carbohydrate, lipid, and protein metabolism. Its activity was enhanced in chickens with increased weights of the abdominal fat pad (36), which supports the hypothesis that the pathway is highly associated with fat content. In our study, the TCA cycle was the most enriched pathway involved in the carbohydrate metabolism of HWS. Gluconeogenesis, a metabolic pathway involved in the production of glucose from specific noncarbohydrate carbon substrates was greater in HWS than LWS. This is in accordance with the observation that gluconeogenesis was more active in the intestinal microbial communities of rabbits with high finishing weight (33). Our results showed that cecal microbiota may have an important role for the host in extracting energy from its diet. Therefore, the shifting of cecal microbes induced by artificial selection may functionally contribute to differences in body weight.

In addition to its effect on the microbiota, artificial selection could directly affect the evolution of the other component of the hologenome, the genome of the host. The HWS and LWS lines that were generated by artificial selection from a common founder population provided a model to explore the evolutionary mechanisms of the hologenome. Previously, using the same chicken model, several studies were carried out to identify the effect of selection on the host genome. Many regions of differentiation between HWS and LWS were identified to contribute to body weight (37–41). Rubin et al. reported a deletion located on the first exon of the gene *SH3RF2* that was fixed in the HWS chickens and occurred at a low frequency in the LWS chickens (37). *SH3RF2* expressed in the hypothalamus of LWS chickens, but not in that of HWS chickens, resulted in a genetic defect in hypothalamic appetite regulation (37). In addition, more recently, several adaptive selective sweeps of the host genome in response to body weight selection were identified (39–41). With more detailed analyses of the selective sweeps, a region located in the *growth1* QTL was reported to be close to fixation in LWS but showed multiple haplotypes segregated in HWS. These findings demonstrated that the host genome was adaptively altered during long-term divergent selection for body weight. However, these known associated regions can only explain part of the variation in body weight (41). Therefore, we studied the differential DNA methylation, mRNA expression, and microRNA profiles in the ceca of HWS and LWS and investigated the effect of the host epigenetic profile on the body weight under long-term artificial selection.

Metabolites, such as SCFAs produced by the intestinal microbiota, induced epigenetic alterations in the intestine of the host (42, 43). This was because the DNA methyltransferases were highly sensitive to the availability of nutrients that can be influenced by the metabolic activities of the present microbial communities in the intestine (44). For example, some of the major SCFAs, such as butyrate, can affect DNA methylation processes through inducing phosphorylation of *MAPK1* (45, 46). In our study, bacteria that are known for butyrate production (46), such as *Faecalibacterium* and *Subdoligranulum*, were highly enriched in the intestine of HWS. This may be partly responsible for the 4,779 DMR throughout the HWS and LWS genomes. We speculate that epigenetic modification is another way for microbes to interact with the host genome during the evolution of the holobiont. However, more in-depth research is necessary to elucidate the complex relationships between intestinal microbiota and host genome methylation.

Additionally, in the cecal transcriptome analysis, four GO terms related to the functions of cAMP were enriched by DEGs. cAMP, a second messenger involved in intestinal epithelial cell homeostasis (47), can regulate the transcription of genes involved in glucose and lipid metabolism. Gluconeogenesis in the intestine is mediated by glucose-6-phosphatase which is transcriptionally regulated by cAMP levels in the enterocytes (48). Lipid metabolism in the intestine is also regulated by cAMP via the cAMP-responsive element-binding protein H (48). Therefore, 5 DEGs, such as *ADCY1* and *DRD4*, involved in cAMP synthesis and metabolism may affect the functions of lipid metabolism and gluconeogenesis in the ceca and indirectly influence the growth and development of chickens. Here, the expression of *KNG1*, *TNNC2*, *MYBPC3*, *CASQ2*, *MYL2*, and *KCNB2* were greater in the ceca of HWS relative to LWS. These 6 genes have been annotated to GO terms of muscle contraction-related functions that were significantly enriched and, thus, suggest that the ceca of HWS and LWS may have influenced different patterns of muscle contractions. This is because muscle contractions of the intestine contribute to the coordinated digestion, absorption of food and nutrients, and, thus, may partly be indirectly associated with the differences in body weight of HWS and LWS.

In the present study, the expression of several DEGs of the ceca was significantly associated with microbes that were differentially enriched in HWS and LWS. The shifting of the genes and cecal microbes may have both contributed to the variation of body weight. We observed that *IGF2BP1* was most significantly upregulated in HWS. *IGF2BP1*, a receptor of *IGF2*, is key in regulating the translation of *IGF2* (49), which has important roles in cell proliferation and differentiation, muscle development, and bone growth (50). A greater expression of *IGF2BP1* in ducks, caused by a genetic mutation, increased body size by 15% and feed efficiency by 6% (51). *IGF2BP1* has also been associated with growth or fat deposition traits in chickens (52), goats (53), and mice (54). Dwarfism and impaired intestine development have been demonstrated in *IGF2BP1*-deficient mice (55). The deletion of *IGF2BP1* in intestinal epithelial cells was shown to compromise barrier function and ameliorate experimental colitis in mice, thus suggesting a role for *IGF2BP1* in maintaining intestinal homeostasis (56, 57). More recently, Wang et al. confirmed the association of IGF2BP1 with chicken body weight by large-scale genomic screening and functional studies (58). However, the way *IGF2BP1* affects the weight of the host is still poorly understood. In our study, the high expression of *IGF2BP1* in HWS may be regulated by both a DEM, gga-miR-2128 and a DMR located in CpG island and whose distance to TSS of *IGF2BP1* was only 388 bp. Correlation analyses of *IGF2BP1* expression and cecal microbes showed that *IGF2BP1* was significantly associated with microbes, such as *Lactobacillus*, more specifically, Lactobacillus salivarius, which were highly abundant in HWS. As a growth promoter, Lactobacillus salivarius additives could increase the size and weight of young animals, such as chickens (59, 60), mice (61), and pigs (62). Meat-type chickens fed a Lactobacillus salivarius mixture isolated from their intestines increased populations of beneficial bacteria, such as *Lactobacillus* and *Bifidobacterium* in the intestine and, thus, increased body weight (59). Overall, results suggest that *IGF2BP1* collaborated with these microbes to influence holobiont traits and support the theory that holobiont phenotypes are affected by both the host and its associated intestinal microbiota.

Long-term divergent selection for 56-day body weight in chickens has not only altered the composition of intestinal microbiota, but also modified host epigenetics, genes, and microRNA profiles of the ceca. The structure of the cecal microbiome was distinct from those of other segments of the GI tract. Functional analyses of cecal microbiota revealed that pathways, such as carbohydrate and energy metabolism, differed significantly between the HWS and LWS lines, indicating an important role that the ceca played during the 56 generations of divergent selection for body weight. Furthermore, several cecal DEGs, such as *IGF2BP1*, were significantly positively correlated with the abundance of cecal microbes, suggesting that *IGF2BP1* mediated the interaction between the host and its intestinal microbes. Overall, our findings demonstrate that the host and its intestinal microbiome both contribute to the evolution of the holobiont and provide evidence to support that the intestinal microbiome cooperates with the host for adaptation as a hologenome.

## MATERIALS AND METHODS

### Animals and sampling.

All procedures were approved by the Virginia Tech Institution Animal Care and Use Committees (IACUC 18-151). The chickens utilized in this experiment were from generation 56 of the Virginia high (HWS) and low (LWS) body weight lines. These lines were established in 1957 from a common founder population obtained by crossing 7 partially inbred lines of White Plymouth Rock chickens (63) (Fig. 1B). Since then, they have been subjected to divergent selection for high or low 56-day body weight, respectively. After 56 generations of selection, the high and low weight lines differed approximately 15-fold. The 56-day body weights (mean ± SD) of HWS females and males were 1510 ± 88 g and 1848 ± 160 g, respectively. For LWS, the values (mean ± SD) were 92 ± 26 g for females and 130 ± 23 g for males. (Fig. 1B). At 245 days of age, 10 HWS and 10 LWS chickens were euthanized by cervical dislocation, and the intestinal contents of the duodenum, jejunum, ileum, ceca, and colon were collected and stored at 4°C with long-term storage at −70°C. In total, 85 high-quality microbial genomic DNA samples (14 duodena, 11 jejuna, 20 ilea, 20 ceca, and 20 cola) were obtained from these intestinal contents (Table S1). In addition, 20 cecal tissues collected from 10 HWS chickens and 10 LWS chickens were immediately flash-frozen in liquid nitrogen and stored at −70°C until further use for DNA and RNA extractions. The husbandry conditions of sampled chickens and protocols of DNA and RNA extraction are presented in Text S1.

10.1128/mSystems.01261-21.1 TEXT S1 Supplemental methods and results. Download Text S1, DOCX file, 0.03 MB. Copyright © 2022 Zhou et al. 2022 Zhou et al. https://creativecommons.org/licenses/by/4.0/ This content is distributed under the terms of the Creative Commons Attribution 4.0 International license.

### Multiomics sequencing summary.

We performed the 16S rRNA gene sequencing for the 85 intestinal (duodenum, jejunum, ileum, ceca, and colon) microbiota samples (Table S1). Twenty microbial genomic DNA samples from ceca were used to construct whole-genome shotgun sequencing libraries (Nextera DNA Library Preparation kit, Illumina). The mRNA sequencing was performed for 20 cecal mRNA samples using the Illumina Truseq RNA sequencing kit. In addition, 20 cecal RNA samples were extracted for small RNA sequencing based on Illumina TruSeq Small RNA Sample Preparation protocol. Genome-wide DNA methylation patterns for 20 cecal DNA samples were quantified using MBD protein capture (MethylMiner kit, Invitrogen). The detailed protocols of the sequencing technology are provided in Text S1.

### Intestinal bacterial 16S rRNA data analysis.

The 16S rRNA raw data were filtered as in our previous study (64). Briefly, barcodes and sequencing primers were trimmed from sequencing reads. Trimmed and assembled sequences were uploaded to the QIIME (v.1.9) (65) and MG-RAST (v.3.6) (66) pipelines for further analysis. The trimmed sequence of each sample was compared to the Greengenes databases (v. 13.8) using the best hit classification option to classify the abundance in QIIME (v.1.9) (65) and MG-RAST (v.3.6) (66), respectively. For QIIME, data on the OTU level were generated using the uclust script (http://qiime.org/scripts/pick_otus.html), and then according to these OTUs, QIIME automatically generated phylum to genus level data for the different intestinal segments and genotypes. To ensure an even sampling depth, data were rarefied to 25,840 sequences per sample (the lowest read number in samples) for the subsequent diversity analyses. Alpha diversity (Shannon, ACE, Simpson, and Chao1) was calculated by mothur (v.1.30) (67). Beta diversity was analyzed using unweighted UniFrac distance and visualized by Canonical analysis of principal coordinates (CAP) using the R package “BiodiversityR” (v.2.8-4, https://cran.r-project.org/web/packages/BiodiversityR/index.html). Linear Discriminant Analysis (LDA) effect size (LEfSe) (v.1.0) (68), an algorithm to robustly identify features that were statistically different among biological classes, was applied to identify microbes from different taxa between lines using the default parameters (LDA > 2, *P* < 0.05). Correlation analyses among different intestinal segments were conducted in Excel using the Pearson method.

### Microbial metagenome data analysis.

After sequencing, a data cleaning process applied to each sample removed low-quality and low-compositional-complexity reads. On average, 37.9 million reads per sample were used in the analysis. Quality-filtered reads were submitted to MG-RAST (v.3.6) (66) and compared to the Kyoto Encyclopedia of Genes and Genomes (KEGG) database (v.88.1) (69). We followed the criteria of the ‘all annotation’ option for functional annotation with a maximum E value cutoff of 10^−5^, a minimum percent identity cutoff of 90%, and a minimum alignment length cutoff of 20 amino acids. Functional pathways, which had a relative abundance greater than 0.1%, for at least 5 samples, were selected for further analysis. Normalization was performed based on the relative abundance of each functional pathway within each sample. Differential analysis was calculated by STAMP (v.2.0) (70) between HWS and LWS for normalized abundance data of each functional pathway using the two-side Welch’s *t* test and false discovery rates (FDR) correction (FDR < 0.05). Taxonomic assignments at the species level for the metagenomic data were carried out using Metaphlan2 (v.2.7.0) (71).

### mRNA sequencing data analysis.

To obtain clean data, adapter trimming and removal of low-quality reads (reads with ambiguous bases N and Q < 20) and poly (A/T) and short sequences (<50 bp) were performed. The quality of filtered reads was then analyzed using FastQC (v. 0.11.4, http://www.bioinformatics.babraham.ac.uk/projects/fastqc/). Then, each clean read was mapped to the Galgal4 reference genome (http://hgdownload.cse.ucsc.edu/goldenPath/galGal4/bigZips/) using TopHat2 (v. 2.1.0) (72). To estimate the expression level of mapped genes, read counts of annotated unigenes were summarized by HTSeq (v. 0.6.1) (73). The RPKM (reads per kb of exon model per million mapped reads) values were calculated based on the number of reads that mapped to each gene and the length of the gene (74). The DEseq program (v.1.18.0, https://www.bioconductor.org/packages/3.0/bioc/html/DESeq.html) was used to analyze differential expression genes (DEG), and those genes with *P* < 0.05 and >2-fold change in value were considered significantly different. The Gene Ontology (GO) (http://www.geneontology.org/) and KEGG enrichment (http://www.genome.jp/kegg/) analyses were performed by the Database for Annotation, Visualization, and Integrated Discovery (DAVID) (v.6.8) (75).

### Small RNA sequencing data analysis.

To obtain clean reads, the raw reads generated from small RNA sequencing were processed by removing the low-quality sequences and trimming the adapters. Then, clean reads, ranging from 15 to 35 bp, were used for further analysis. After identifying the unique reads, each was mapped to Galgal4 reference genome using Bowtie (v. 22.1.0) (76) and BLAST (v.2.2.28, https://blast.ncbi.nlm.nih.gov/Blast.cgi), and they were searched against the ncRNA database Rfam (v.10.1, http://rfam.xfam.org/) to obtain the distribution of reads in the genome and ncRNA annotation. The unique reads were first matched against the chicken miRNA database in miRbase (v. 20.0) (77) to confirm known chicken miRNAs, then against the databases of other species in miRbase to identify those miRNAs homologous to known miRNAs in other species. Then, the program mireap (v.0.2, http://sourceforge.net/projects/mireap/) was used to predict potentially novel chicken miRNAs and their precursors. The differentially expressed miRNAs (DEM) were identified on the base of a fold change either ≥ 2 or ≤ 0.5 and *P* < 0.05 using the DEseq program (v.1.18.0). After we obtained the DEMs, miRanda software (v.3.3a) (78) was used to predict the targets genes of DEMs. Then GO and KEGG analyses of the target genes were performed using DAVID (v.6.8) (75).

### Whole-genome methylation sequencing data analysis.

After the raw data obtained from Illumina sequencing were processed to filter out reads containing adapters, unknown or low-quality bases quality control was performed using FastQC (http://www.bioinformatics.babraham.ac.uk/projects/fastqc/). Clean reads were then aligned to the Galgal4 reference genome by Bowtie (v. 22.1.0) (76). The distribution of the aligned data in different components of the genome and chicken chromosomes were analyzed using RseQC (79) (v. 2.3.7). Then, the bam files containing unique aligned reads were performed to detect peaks using R package MEDIPS (v.1.22, http://www.bioconductor.org/packages/release/bioc/html/MEDIPS.html). The default parameters of MEDIPS were applied as follows: uniq = TRUE, extend = 300, shift = 0, window size = 100. To identify the differentially methylated regions (DMR), MEDIPS runs an edgeR analysis (80), which is an empirical Bayes method. The FDR was used for multiple test corrections. The threshold utilized was 0.1. Significant regions (FDR < 0.1) were used for downstream analysis. After confirming genomic windows that showed a significant differential coverage between conditions, the DMRs were annotated with HOMER software (v.4.10, http://homer.ucsd.edu/homer/). HOMER provided detailed information about the location of regions, including exon, intron, 5’UTR, 3’UTR, TSS, TTS, and intergenic. Genes that overlapped with DMRs were identified.

### Correlation analyses between microbiota and host.

To measure relationships between DEGs and cecal microbes, correlation analyses were carried out using the Pearson method provided by R packages psych (v. 1.8.12, https://CRAN.R-project.org/package=psych). Test analyses for correlation coefficients were performed by corr.test using the FDR method for multiple tests. The upregulated DEGs with FDR < 0.01 in HWS and LWS were the inputs for the correlation analyses with all cecal microbes annotated at each level. Those genes and microbes with |r|>0.5 and *P* < 0.01 were used to construct the network. The method used for correlation analyses between DEMs and microbes was the same as that used for DEGs.

### Data and materials availability.

The sequencing data of small RNA, mRNA, DNA methylation, and metagenome analyzed during this study are available in the Sequence Read Archive (https://www.ncbi.nlm.nih.gov/sra) with the accession codes PRJNA601115. The 16S sequences are publicly available in MG-RAST (http://www.mg-rast.org/) under the project name “Chicken_HW_LW_16S”. All other relevant data are available from the authors.

10.1128/mSystems.01261-21.5 FIG S4 The interaction network between significantly differentially expressed miRNAs and genera in the ceca, (A) up-regulated miRNAs in HWS with genera, (B) down-regulated miRNAs with genera. Download FIG S4, TIF file, 2.8 MB. Copyright © 2022 Zhou et al. 2022 Zhou et al. https://creativecommons.org/licenses/by/4.0/ This content is distributed under the terms of the Creative Commons Attribution 4.0 International license.
