# Supplementary figures and images for: Intestinal Microbiota and Host Cooperate for Adaptation as a Hologenome
Source: mSystems. 2022 Jan 11;7(1):e01261-21. doi: 10.1128/msystems.01261-21 (PMC8751389; doi:10.1128/msystems.01261-21)

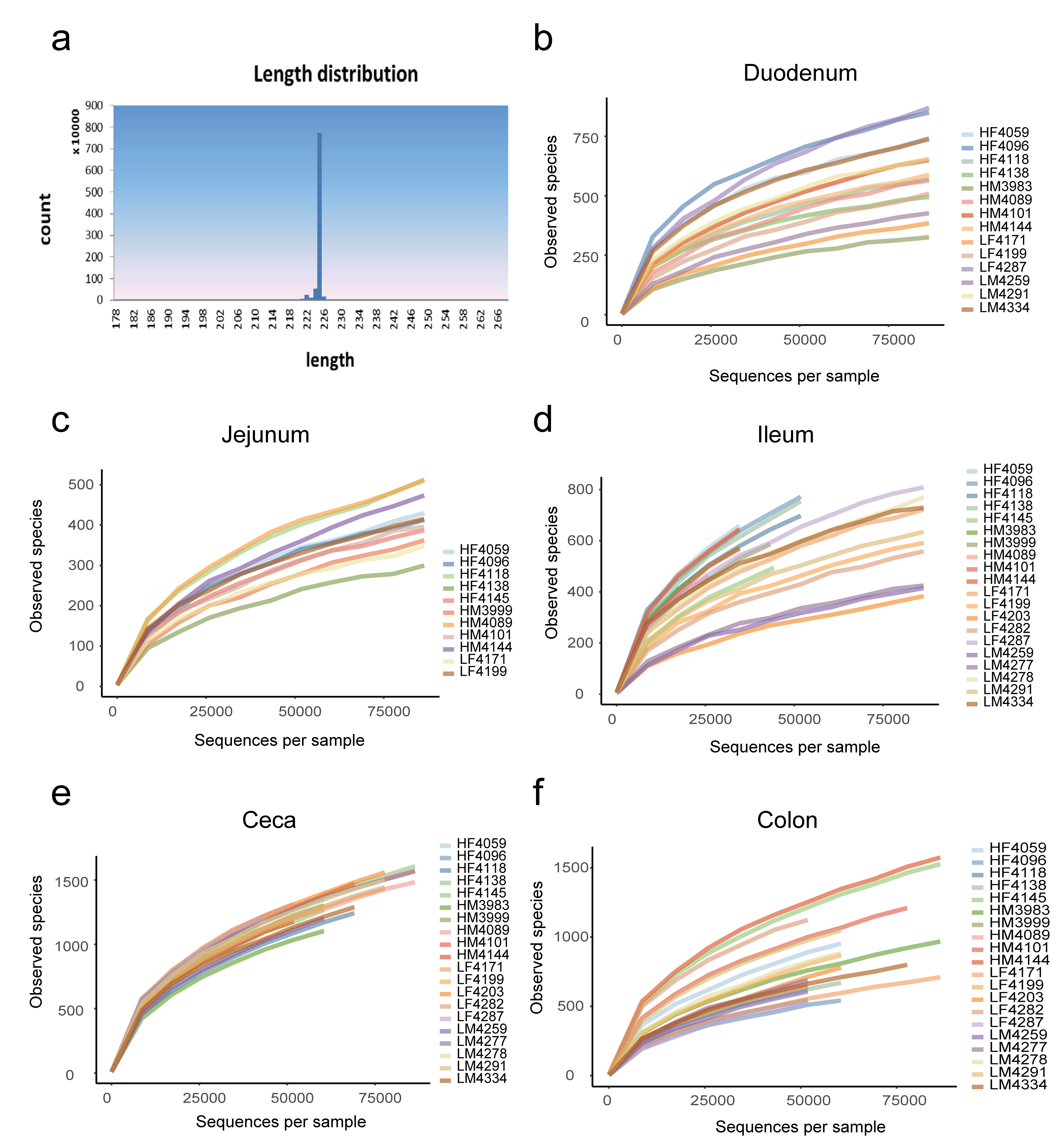

Supplement: FIG S1 [file msystems.01261-21-sf001.tif]

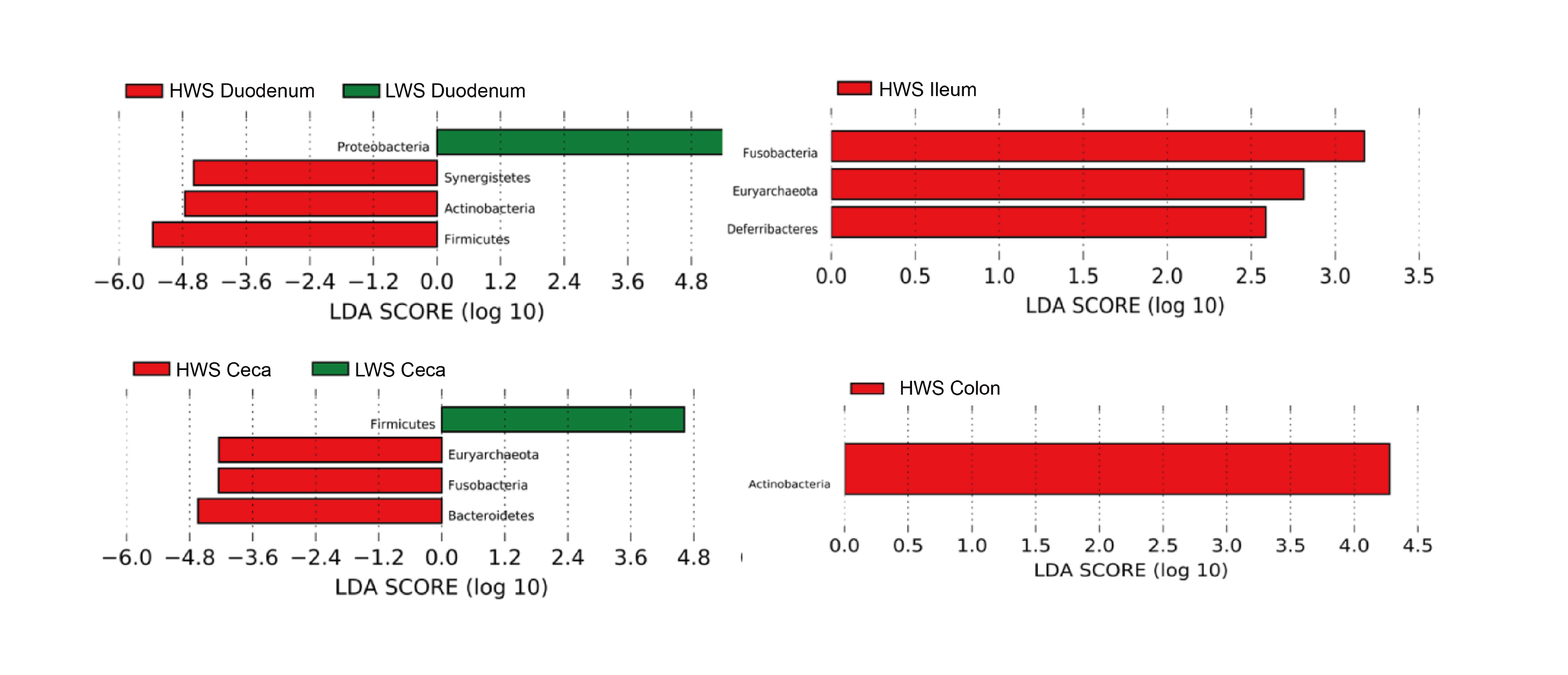

Supplement: FIG S3 [file msystems.01261-21-sf003.tif]

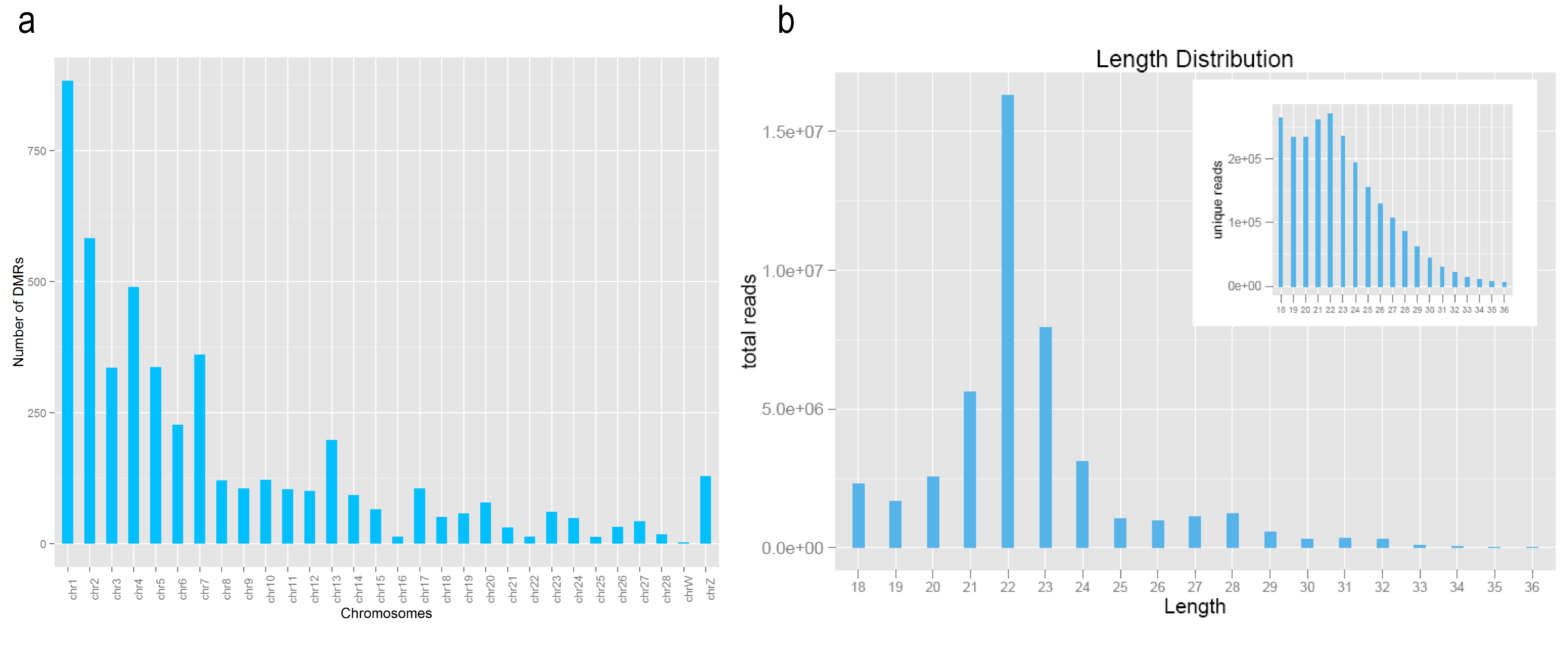

Supplement: FIG S2 [file msystems.01261-21-sf002.tif]

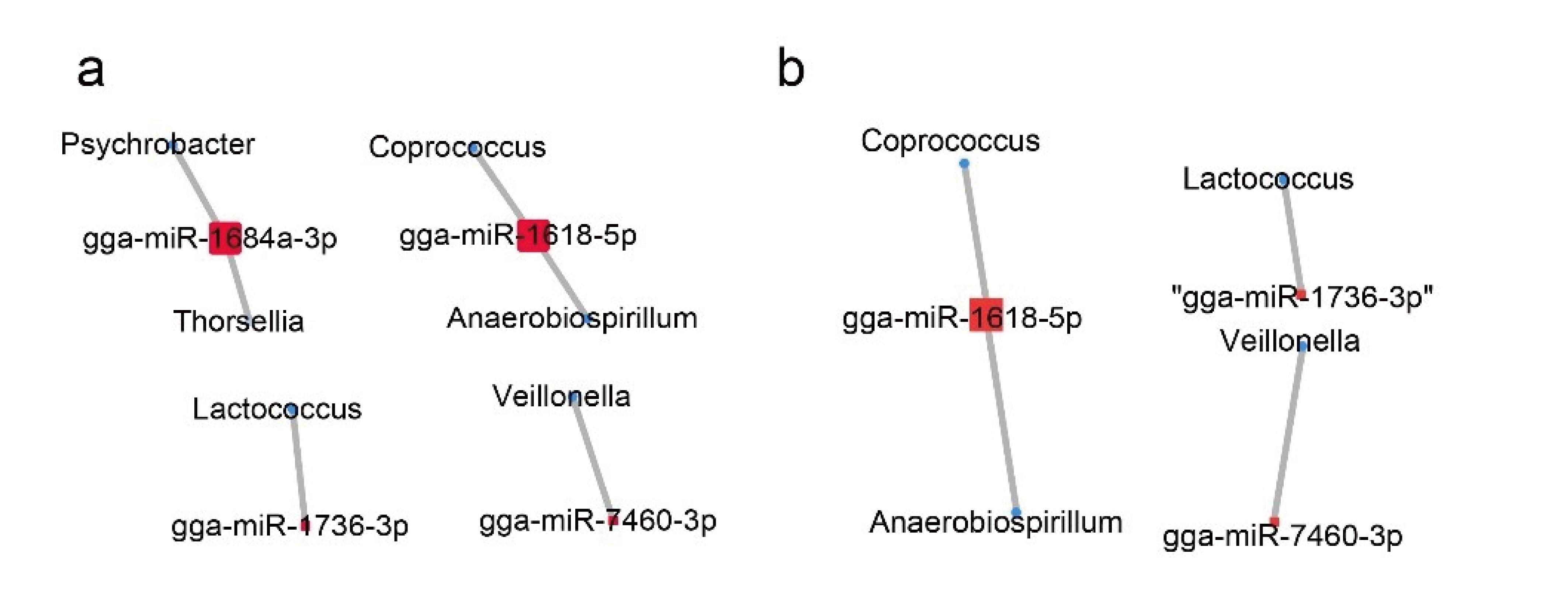

Supplement: FIG S4 [file msystems.01261-21-sf004.tif]
