# Supplementary material for: Intestinal Microbiota and Host Cooperate for Adaptation as a Hologenome
Source: mSystems. 2022 Jan 11;7(1):e01261-21. doi: 10.1128/msystems.01261-21 (PMC8751389; doi:10.1128/msystems.01261-21)
Supplement: TEXT S1 [file msystems.01261-21-s0001.docx]

Supplementary Materials for

- **Intestinal microbiota and host cooperate for adaptation as a hologenome**

Hao Zhou^1,^ ^†^, Lingyu Yang^1,^ ^†^, Jinmei Ding^1^, Ronghua Dai^1^, Chuan He^1^, Ke Xu^1^, Lingxiao Luo^1^, Lu Xiao^1^, Yuming Zheng^1^, Chengxiao Han^1^, Fisayo T. Akinyemi^1^, Christa F. Honaker^2^, Yan Zhang^3,*^, Paul B. Siegel^2,*^, He Meng^1,*^

*Corresponding authors

Yan Zhang: yzhang1@carilionclinic.org

Paul B. Siegel: pbsiegel@vt.edu

He Meng: menghe@sjtu.edu.cn

Supplementary Text

Supplementary materials and methods

**Sampled chickens.** All chickens from the 56^th^ generation of the high (HWS) and low (LWS) weight lines were hatched on the same day and were from parents of the same age. Chicks from each line were penned separately, in the same building. At 19 weeks of age, they were transferred to individual cages with gently sloping wire floors (dimensions of 48l X 28w X 38-43h cm). The photoperiod was 14:10 hour light:dark. Chickens were fed starter (0-8 weeks), developer (8-19 weeks), and breeder (thereafter) antibiotic-free corn-soybean diets in mash form. Chickens were from common aged parents that were housed in the same manner as their progeny.

**DNA extraction.** Microbial genomic DNA was extracted from intestinal content samples following kit protocol of a TIANGEN DNA stool mini kit (TIANGEN, cat#DP328). The DNA from cecal tissues of each of 20 individuals (10 per line) was extracted with a PureLink^TM^ Genomic DNA Mini Kit and its quality verified by 1% agarose gel electrophoresis.

**RNA extraction.** Total RNA was extracted from cecal tissue using TRIzol® reagents following the manufacturer’s protocols (Invitrogen, Carlsbad, CA). Degradation and contamination of the total RNA was detected on 1% agarose gels. The purity of the total RNA was assessed using a NanoPhotometer® spectrophotometer (IMPLEN, CA). The integrity was confirmed with the Agilent 2100 Bioanalyzer (Agilent Technologies) with clear characteristic peaks at 28S and 18S. All samples measured had a RIN value above 8.0. The RNA concentration was checked with a Qubit® RNA Assay Kit in a Qubit® 2.0 fluorometer (Life Technologies, CA), and samples were stored at −80°C for further analysis.

**Gut bacterial 16S rRNA gene sequencing.** The V4 hypervariable regions of the 16S rRNA were PCR amplified from the microbial genomic DNA, using barcoded fusion primers (forward primer: 5’-AYTGGGYDTAAAGNG-3’; reverse primer: 5’-TACNVGGG TATCTAATCC-3’). The PCR program was as follows: initial denaturation at 94°C for 5 minutes, 94°C denaturation for 30 seconds, 50°C annealing for 30 seconds, and 72°C extension for 30 seconds, repeated for 27 cycles with a final extension at 72°C for 7 minutes. The PCR products were purified using a QIAGEN quick Gel Extraction Kit (QIAGEN, cat# 28706). Barcoded V4 PCR amplicons were sequenced by an Illumina Miseq platform. Amplification of the V4 regions of 16S rRNA genes and sequencing services were provided by Shanghai Personal Biotechnology Co., Ltd. (Shanghai, China).

**Metagenomic sequencing****.** Microbial genomic DNA for each of the 20 individual samples from HWS and LWS was used to construct whole microbial genome sequencing libraries with insert sizes of 300 and 400 bp (Nextera DNA Library Preparation kit, Illumina). Each library was sequenced by high-throughput sequencing at 2 × 100 bp using the Illumina HiSeq 2000 (Illumina, USA).

**mRNA sequencing of** **cecal tissues.** The 20 mRNA libraries of HWS and LWS extractions were constructed following the manufacturer’s instructions (TruSeq RNA Sample Prep Kit, Illumina). The mRNA was purified from the total RNA using magnetic oligo (dT) beads. Fragmentation buffer was used to cut mRNA into approximately 155 bp fragments. After double strand cDNAs were synthesized, end repair and poly-adenylation were performed, and the cDNA was connected with sequencing adapters before PCR amplification. Enriched cDNA with suitable lengths were purified and used for subsequent analyses. Library quantification was performed by Pico green and a fluorescence spectrophotometer (Quantifluor-ST fluorometer, Promega; Quant-iT Pico Green dsDNA Assay Kit, Invitrogen). The library quality was confirmed by the Agilent 2100 Bioanalyzer (Agilent 2100 Bioanalyzer, Agilent; Agilent High Sensitivity DNA Kit, Agilent). Finally, the cDNA libraries were sequenced with paired-end 2 × 100 nt multiplex on an Illumina HiSeq 2000 platform.

**Small RNA sequencing of cecal tissues.** The RNA samples for small RNA sequencing were the same as those for mRNA-Seq. The RNA libraries for small RNA-Seq were constructed following the manufacturer’s protocols. Briefly, after the 3’ and 5’ adapters were ligated to the total RNA, the ligated RNA was reverse-transcribed to cDNA. Then, the cDNA, which had adapters was amplified by PCR. The PCR products with suitable length were isolated from polyacrylamide gels to construct the small RNA libraries. The library preparations were sequenced on an Illumina Miseq system by Shanghai Personal Biotechnology Co., Ltd. (Shanghai, China).

**Whole genome methylation sequencing** **of cecal tissues.** Genomic DNA of 5 cecal tissues from 5 individuals of a sex-line group was extracted using the PureLin™ Genomic DNA Mini Kit (Invitrogen, A1120), following the manufacturer’s protocols. The quality of the DNA was then evaluated by agarose gel electrophoresis and a microplate reader (Eppendorf, Germany). The MBD-Seq method was used to identify methylated DNA regions. The MethylMiner methylated DNA enrichment kit (Invitrogen) was used to obtain DNA containing methylated CpGs. First, the DNA was fragmented to an average length of 300–400 bp by pumping high-pressure nitrogen gas and using agarose gel to visualize the size of the resulting segments. Simultaneously, the DynabeadsRM-280 Streptavidin and the MBD-Biotin protein were mixed for about an hour to obtain coupled MBD-beads for capturing methylated DNA. Second, 3'-5' exonuclease and DNA polymerase were used for the end repair of the fragmented methylated DNA. Then, a 3’ A was added using DNA Polymerase I, Large (Klenow) Fragment (NEB, M0210L). Also, a pair of Illumina adaptors was ligated to the repaired ends by T4 ligase (Promega, M1801). Filtration in a 2% agarose gel was used to select fragments (DNA plus adaptors) from 200 to 500bp. DNA templates were PCR-amplified with Phusion high-fidelity polymerase (NEB, M0530S). After purification, methylated DNA was captured by prepared MBD-beads. The captured DNA was precipitated, which was eluted by high-salt Elution Buffer using ethanol. Finally, the library was quantified with a Quant-iT PicoGreen dsDNA Assay Kit (Invitrogen, USA) using an Agilent 2100 Analyzer (Agilent Technologies, USA). Following the manufacturer’s protocols, the MBD library was subjected to high-throughput sequencing with an Illumina HiSeq2000 platform to generate paired-end 100-bp reads by the Shanghai Personal Biotechnology Co., Ltd. (Shanghai, China).

Supplementary results

**Basic analysis of RNA sequencing data.** The RNA-Seq generated a total of 926,441,208 and 998,664,168 raw reads from the HWS and LWS lines, of which, 692,887,916 (74.79% of raw reads) and 732,263,156 (73.32% of raw reads) cleaned reads, respectively, were obtained after the low-quality reads and adapters were filtered (see Table S19 at https://doi.org/10.6084/m9.figshare.16830736.v1). The bowtie mapped the clean reads in reference to the chicken genome and showed that 80.48% of HWS and 79.30% of LWS reads could be uniquely aligned. In addition, an average of 68.27% and 66.93% reads in HWS and LWS could be mapped to genes, respectively. A total of 15,509 genes from the two lines were identified.

**Basic analysis of miRNA profiles.** Of the 97,402,212 clean reads obtained from each of the 20 small RNA libraries (see Table S20 at https://doi.org/10.6084/m9.figshare.16830736.v1), the length of most reads were between 22-24 nt (Fig S2b). Of them, 78% could be mapped to the reference chicken genome. These reads then were used for miRNA annotation.

**Data collation of MBD-Seq.** MBD-Seq generated 575,675,384 and 681,044,974 raw reads from HWS and LWS, respectively. Of these, 468,049,258 and 475,915,506 were clean reads (average length 100 bp), respectively. On average, 80.42% of unique aligned reads were aligned on the chicken genome. Detailed information of mapping rate for each sample is described in Table S21 [https://doi.org/10.6084/m9.figshare.16830736.v1].

**Correlation analyses of DEMs and gut microbiota in ceca.** Correlation analyses showed that 4 of 6 up-regulated miRNA in HWS were significantly associated with 6 genera (Fig. S4a). Among them, *Lactobacillus* was significantly enriched in their ceca. It was connected to miR-1736-3p with an r value of 0.60. In addition, 3 of 6 up-regulated miRNAs in LWS were linked to 4 genera, with 3 of them also connected to up-regulated miRNA in HWS. These were *Coprococcus, Lactobacillus,* and *Anaerobiospirillum* (Figure S4b).
